# Maternal depression is associated with altered functional connectivity between neural circuits related to visual, auditory, and cognitive processing during stories listening in preschoolers

**DOI:** 10.1186/s12993-020-00167-5

**Published:** 2020-04-27

**Authors:** Rola Farah, Paige Greenwood, Johnathan Dudley, John Hutton, Robert T. Ammerman, Kieran Phelan, Scott Holland, Tzipi Horowitz-Kraus

**Affiliations:** 1 Educational Neuroimaging Center, Faculty of Education in Science and Technology, Faculty of Biomedical Engineering, Technion, Haifa, Israel; 2 Reading and Literacy Discovery Center, General and Community Pediatrics, Cincinnati Children’s Hospital Medical Center, Cincinnati, OH USA; 3 Cincinnati Children’s Hospital Medical Center and University of Cincinnati College of Medicine, Cincinnati, OH USA; 4 The Permanente Medical Group, San Rafael Pediatrics, San Rafael, CA USA; 5 Department of Physics, University of Cincinnati, Cincinnati, OH USA; 6 Pediatric Neuroimaging Research Consortium, Cincinnati Children’s Hospital Medical Center, Cincinnati, OH 45229-3039 USA

**Keywords:** Maternal depression, Childhood development, Narrative comprehension

## Abstract

**Background:**

Maternal depression can influence the early activity of a mother reading stories to a young child, as depressed mothers are less likely to read to their children. Here, maternal depression association to neurobiological circuitry of narrative comprehension, visualization, and executive functions during stories listening was examined in 21 4-year-old girls and their mothers. Maternal depression scores were collected from the mothers, and functional MRI during stories listening was collected from the children.

**Results:**

Increased maternal depression was related to decreased functional connectivity between visualization and auditory regions and increased connectivity between the right visual cortex and dorsolateral prefrontal cortex in the children.

**Conclusions:**

This study highlights the need to monitor maternal depression and provide interventions to ensure positive linguistic outcomes in children.

## Key points

- Previous research has indicated that maternal depression plays a role in childhood development (i.e., in executive functions) and that depressed mothers speak differently to their children.
- The current study explored the neurobiological underpinnings of maternal depression and child language development.
- Maternal depression was related to decreased functional connections between visualization and auditory regions and increased connectivity in frontal and visual regions in the children during stories listening.
- Maternal depression should be observed during child-wellness office visits so that interventions such as dialogic reading could be implemented to ensure cognitively stimulating interactions between mother and child.

## Background

### Child language development: critical period and components

Early childhood is a critical period for development. The young brain learns the sounds and meaning of language. Extensive synaptogenesis and pruning occurs from birth until the age of 7 years, when children learn the phonological and grammatical characteristics of the language they are exposed to [1], and is ongoing into adolescence in the frontal cortices, especially in the dorsolateral prefrontal cortex (DLPFC) [2]. Young children use a set of innate human-specific skills, particularly auditory word recognition and syntactic and semantic processing, when listening to linguistic stimulation [3]. As found by functional magnetic resonance imaging (fMRI), these linguistic abilities rely on phonological processing supported by the superior temporal gyrus (STG, BA 22) and the angular gyrus (AG, BA 40, 39), as well as semantics and syntax engaging the inferior frontal gyrus (IFG, BA 44, 45). The ability to comprehend language also relies on abilities that are not specific to language, such as executive functions, supported by frontal regions (BA 10, 6, 46, 9, 8). Each of these regions proves instrumental in the development and usage of the mentioned linguistic functions [4].

An additional cognitive ability essential for language processing is visualization [5]. Visualization is the ability to create a visual representation of verbal stimulation and has been found to be related to improved narrative comprehension [6] and reading comprehension [7]. The role of BA 17, 18 in visual imagery or visualization has been previously determined (see [8]). Increased literacy environment (i.e., more books in the household, more hours read to the child) in children ages 3–5 years is related to increased activation in visual processing regions during stories listening [6]. The presumption was that children with increased environmental stimulation are able to imagine the stories better by relying on visual processing regions. These findings were supported by our additional findings in the same age population, 4-year-old children who listened to stories while their future reading network was defined based on previous studies [9]. We demonstrated that stories listening in 4-year-old children “fit into” their future reading network and suggested that recruitment of regions related to visual imagery during the process of listening to stories will support future reading acquisition.

### Maternal depression and child language development

One of the most influential environmental components in a child’s life is his or her primary caregiver, typically the mother. Mother–child interaction may be affected by the mental state of the mother [10]. Maternal depression is manifested through clinically elevated depressive symptoms and is prevalent in low-income mothers. Ammerman and colleagues reported that 44.3% of 806 mothers exceeded a clinical cutoff for a depression screening on at least one of two administrations during the 1st-year postpartum Ammerman, Putnam [11]. An examination of the relationship between depression and parenting indicated decreased nurturing and less involvement with the child in depressed compared to non-depressed mothers using the HOME Inventory [12].

Depression in mothers may be associated with an absence of consistent environment and a lack of responsiveness to the child Sohr-Preston [10]. The authors suggested that depressed mothers tend to respond negatively to their children and interact with them less, which may lead to decreased cognitive and language outcomes for the child. These mothers tend to use less infant-directed speech, less facial interaction, and less use of “motherese”—a way of speaking that includes higher tones and exaggerated prosody that triggers an infant’s attention [13–16]. Interestingly, such interaction was previously related to the activation of the dorsolateral prefrontal cortex (DLPFC) [2, 17]. Despite the accumulated information of the negative effect of maternal depression levels on interaction with the child and the cognitive and linguistic outcomes, the impact of maternal depression on the developing neural circuitry of the child when listening to stories has not been tested. Revealing the impact of maternal depression on these neurobiological circuits, especially those related to imagination and visualization, may lead to a better understanding of the mechanisms related to decreased linguistic outcomes.

In this study, we sought to determine the neurobiological relationship between maternal depression and child language development, specifically focusing on visualization abilities while listening to stories. fMRI data acquired while 4-year-old girls listened to stories was tested for association with the depression severity of their mothers. Based on several studies demonstrating decreased functional connections between the visual imagery network and cognitive control network or regions in conditions with less engagement vs those with greater engagement [18, 19], we hypothesized that decreased functional connections between the visual processing and cognitive control regions would be found. More specifically we hypothesized that increased maternal depression would be associated with decreased functional connections between visual processing regions and auditory/language regions due to less efficient stories-listening processes in the children of depressed mothers. We also suggested greater functional connections between visual processing regions and cognitive control areas, specifically the DLPFC (per [2, 17]) due to non-automatic visualization processes while listening to stories in girls with mothers exhibiting greater to severe depressive symptoms.

## Methods

### Participants

Children (N = 21, mean age: 48 months, all right-handed girls) and their mothers (N = 21, mean age: 21.57 years) participated in the current study. All children were within the normal range of nonverbal IQ, none had a history of neurological or emotional disorders or attention difficulties, and all were native monolingual English speakers with no contraindications to MRI. Almost half of mothers (47.5%) had less than a college education at the time of the study. We chose to include only girls and their mothers in this study to avoid the confounding influence of sexual dimorphism on child brain-language development and based on previous experience showing greater MRI success rates in girls versus boys in the 4-years-old age group [20, 21]. Written informed consent was obtained from mothers, with verbal assent from the children. Participants were recruited from a longitudinal study of injury prevention in young children of low income, first-time mothers enrolled in a home visiting program [22]. The study received Institutional Review Board approval (CHIP study, Protocol number: 2010-2537).

### Behavioral measures

The performance and verbal subtests from the Wechsler Preschool and Primary Scale of Intelligence (WPPSI) [23] were administered to verify normal nonverbal and verbal IQ, respectively. Verbal ability was assessed by instructing the child to point at the correct picture corresponding to the given verbal cue. Nonverbal ability was assessed by instructing the child to point at the missing part of an incomplete shape. The average of the tests from the WPPSI was 100 with a standard deviation of 15.

To exclude linguistic deficits, phonological awareness was assessed using the Elision subtest from the Comprehensive Test of Phonological Processing (CTOPP) [24]. In this test, the child was requested to omit a sound from an orally given word. The average of the CTOPP test was a scaled score of 10 with standard deviation of 3.

Reading ability of the mothers was assessed by the Test of Word Reading Efficiency (TOWRE), specifically the sight word efficiency and phonetic decoding efficiency subtests [25]. The mothers’ depression levels were assessed using the Beck Depression Inventory-II (BDI-II) [26]. The BDI-II was administered at 12 months postpartum, 24 months postpartum, and at the study visit. This self-report assessment consists of 21 items to assess depression symptoms. A score of 0–13 indicates minimal depression, 14–19 indicates mild depression, 20–28 indicates moderate depression, and 29–63 indicates severe depression [26]. Averages, minimum and maximum values, as well as skewness and kurtosis of the behavioral scores were calculated. To characterize the persistent nature of exposure of children to maternal depression, we calculated the total of each of the three administrations of the BDI–II. This approach has been used to examine the impact of persistent maternal depression on child neurocognitive processes [27].

### MRI procedure

#### Stories-listening task

For the stories-listening fMRI task, a total of five stories were presented in a female voice, one for each 30-s task period (transcripts of a story used can be found in [4] and the audio of stories can be downloaded from https://irc.cchmc.org/software/pedaudio.php). The stories presented were 9, 10, or 11 sentences in length and contained varying syntactic structures and vocabulary appropriate for the preschool level. The sentences were randomized across scans. For contrast with the stories, backwards speech was presented with frequencies of 200–400 Hz for 30-s intervals between each story as a control for sub-lexical auditory processing in the fMRI analysis stage.

#### MRI acquisition and data preparation

The children were acclimated and desensitized to condition them for comfort inside the MRI scanner (see [28, 29] for details). Communication was established between the child and study coordinator through headphones equipped with a built-in microphone. Children were asked to listen to the stories they heard through the headphones. Verbal communication and positive reinforcement were maintained with the child throughout the scan. Scanning was terminated immediately if the child did not wish to continue. Children were not sedated and were awake during the entire duration of the MRI scan, as monitored through the use of an MRI-compatible eye-tracking system (Real-eye, Arrington Research, Inc., Scottsdale, AZ).

MRI scans of the children were acquired using a 3T Philips Achieva MRI system (Philips Medical Systems, Best, The Netherlands) equipped with an Avotec audiovisual system and the eye-tracking system. MRI acquisition included a 3D anatomical brain image for registration of functional data, as well as a fMRI scan during stimulation with a stories-listening task as described here and elsewhere [4, 30]. For fMRI, a time-series of 165 blood-oxygen-level dependent (BOLD)-weighted scans with the following parameters: TR/TE = 2000/38 ms; BW = 125 kHz; FOV = 25.6 × 25.6 cm; matrix = 64 × 64; slice thickness = 5 mm covering the entire brain with 38 slices in the axial plane were acquired with voxel size 3.75 × 3.75 × 5 mm at 2-s intervals during a single run of the stories-listening task.

Data processing was performed using FSL software (fMRI-Brain Software, version 6, Library, Oxford, UK) [31]. Data were processed to correct images for head motion during the time series by using the MCFLIRT routine in FSL. Slice-timing correction, spatial smoothing, and intensity normalization were all applied using the FEAT (fMRI Expert Analysis Tool, version 6.00) modality of FSL for our BOLD analyses, followed by magnetic-field map correction, normalization of all images to the Montreal Neurological (MNI) space, a spatial smoothing with an 8-mm full width at half-maximum (FWHM) Gaussian kernel, and registration to the structural 3D images. In addition, a threshold for excessive motion (> 2 mm) was determined. We used a mutual information cost function for rejecting motion-corrupted frames of fMRI data, as previously described [32]. All data met the criterion of median voxel displacement < 2 mm in the center of the brain.

Following the described preprocessing steps, functional image time-series data were entered into functional connectivity analysis.

#### Functional connectivity analysis

To determine the correlation between maternal depression and the functional connectivity of the child’s processing of imagery, we picked visual processing regions already described in previous studies as participating in imagery processes [33] as the seeds for this analysis. Using the Brodmann area (BA) atlas implemented in the CONN toolbox (http://www.nitrc.org/projects/conn/; [34]), we focused on both bilateral BA 17 and 18 as our seeds-of-interest and correlated the functional connectivity between these seeds and all BAs in the brain with maternal depression scores.

Once the BOLD signal from the background speech was subtracted from the BOLD data from the stories-listening task, analysis of functional connectivity during the stories task was carried out using the CONN toolbox [34]. Normalized bias-corrected *T*_1_ images were generated in SPM (http://www.fil.ion.ucl.ac.uk/spm/) and segmented into gray matter, white matter, and cerebral spinal fluid (CSF). The principle eigenvariate of the BOLD time-courses from white matter and CSF, as well as the six motion-correction parameters were included as regressors-of-no-interest and were detrended from the fMRI time-series data. The data were then band-pass filtered between 0.008 and 0.2 Hz (as recommended [35]), and only data within this range were included in the connectivity analysis in the next step. We then conducted a functional connectivity analysis between seeds placed in the child’s visual processing brain regions and other regions in the brain during stories listening. Finally, we tested these connections for associations with maternal depression level. Data was corrected for multiple comparisons for each ROI.

## Results

### Behavioral measures

Performance and verbal abilities were average to above average in all of the children. Maternal reading ability was in the average to below average range. Maternal depression level was in a mild range. There were 13 mothers with a minimal depression, three with mild depression, two with a moderate depression, and three with severe depression. See Table 1 for details.

**Table 1:** Behavioral measures of children and their mothers

Cognitive ability (average range)	Test	Average (standard deviation)	Min–max	Skewness	Kurtosis
Children					
Phonological awareness (10 ± 3)	Elision, CTOPP (scaled score)	9.00 ± 1.55	7–12	0.892	− 0.694
	Blending words, CTOPP (scaled score)	7.90 (0.94)	6–10	0.204	0.087
	Digit memory, CTOPP (scaled)	7.60 (2.99)	2–14	0.020	− 0.049
Verbal ability (100 ± 15)	Verbal IQ, WPSSI (scaled score)	25.45 (5.99)	14–36	− 0.209	− 0.644
Nonverbal ability (100 ± 15)	Performance IQ, WPSSI (scaled score)	23.10 (7.40)	10–38	− 0.168	− 0.350
General ability (100 ± 15)	Full scale IQ, WPSSI (composite)	91.65 (11.77)	74–120	0.654	0.472
Mothers					
Maternal reading ability (100 ± 10)	Sight word efficiency, TOWRE (scaled)	92.24 (9.67)	71–106	− 0.422	− 0.429
	Phonemic decoding efficiency, TOWRE (scaled)	92.86 (10.49)	71–110	− 0.346	− 0.543
Maternal depression	BDI–II (total average score across 3 years)	15.14 (11.80)	3–45	1.29	0.74

### Neuroimaging measures

#### Functional connectivity of visual processing regions and other regions in the brain during stories listening

**Fig. 1:**
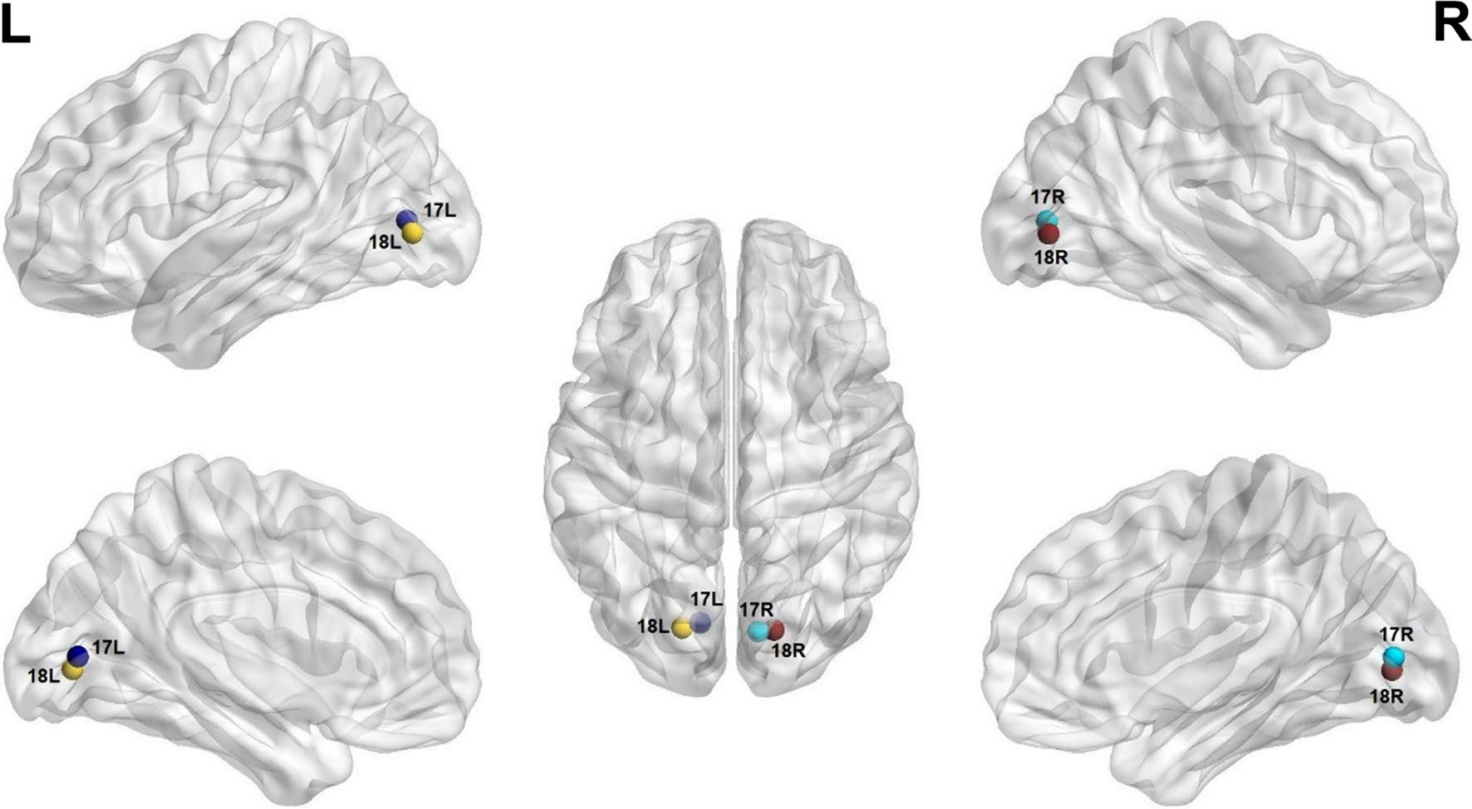
Visual seeds chosen for the analysis; Brodmann areas 17 and 18

Overall, we found positive functional connectivity during the stories-listening task between the child’s left visual cortex (BA 17, BA 18) and regions related to vision (BA 7, 17–19, 30), reward and emotional processing (BA 11, left BA 38), cognitive control (10, 31, left BA 46), word reading (right BA 39), and language (right BA 37). Additionally, positive functional connectivity during the stories task was found between the right visual cortex (BA 17, BA 18) and other visual regions (BA 17–19, 30), language (right BA 37), emotional processing (right BA 38, BA 29, right BA 23), cognitive control (BA 10, 31, left BA 46), reward processing (BA 11), word reading (right BA 39), and memory (right BA 27) (Fig. 1 and listed in Table 2; *P *< 0.05, FDR corrected).

**Table 2:** Target regions exhibiting increased functional connectivity to visual regions during stories listening

Seed	Target (ROI)	Beta	T-value	*P*-value uncorrected	P-value FDR correction
L Primary Visual Cortex (left BA 17)	L Secondary Visual Cortex (BA 18)	0.92	17.78	0.00	0.00
	R Secondary Visual Cortex (BA 18)	0.66	14.83	0.00	0.00
	R Associative Visual Cortex (BA 19)	0.50	12.44	0.00	0.00
	L Associative Visual Cortex (BA 19)	0.46	8.06	0.00	0.000002
	R Primary Visual Cortex (BA 17)	0.54	7.59	0.00	0.000004
	R Orbitofrontal cortex (BA 11)	0.22	5.02	0.000066	0.000917
	L Orbitofrontal cortex (BA 11)	0.18	4.61	0.000169	0.001999
	R Cingulate Cortex (BA 30)	0.20	3.91	0.000861	0.008935
	R Anterior Prefrontal Cortex (BA 10)	0.22	3.46	0.002482	0.022892
	L Cingulate Cortex (BA 30)	0.13	3.37	0.003069	0.025471
	L Temporopolar Area (BA 38)	0.15	3.10	0.005687	0.040299
	L Dorsolateral Prefrontal Cortex (BA 46)	0.14	3.09	0.005826	0.040299
L Secondary Visual Cortex (left BA 18)	L Associative Visual Cortex (BA 19)	0.84	18.04	0.00	0.00
	L Primary Visual Cortex (BA 17)	0.92	17.78	0.00	0.00
	R Associative Visual Cortex (BA 19)	0.75	16.50	0.00	0.00
	R Secondary Visual Cortex (BA 18)	1.02	13.96	0.00	0.00
	R Primary Visual Cortex (BA 17)	0.66	11.48	0.00	0.00
	R Cingulate Cortex (BA 30)	0.47	9.90	0.00	0.00
	L Cingulate Cortex (BA 30)	0.42	9.74	0.00	0.00
	R Dorsal Posterior Cingulate Cortex (BA 31)	0.31	7.42	0.00	0.00004
	L Orbitofrontal Cortex (BA 11)	0.26	5.21	0.000042	0.000359
	L Dorsal Posterior Cingulate Cortex (BA 31)	0.28	5.20	0.000043	0.000359
	R Orbitofrontal Cortex (BA 11)	0.32	5.10	0.000055	0.000413
	R Angular Gyrus (BA 39)	0.18	3.90	0.000886	0.005917
	R Somatosensory Association Cortex (BA 7)	0.24	3.88	0.000927	0.005917
	R Anterior Prefrontal Cortex (BA 10)	0.26	3.82	0.001080	0.006405
	R Fusiform Gyrus (BA 37)	0.19	3.74	0.001293	0.007152
	L Somatosensory Association Cortex (BA 7)	0.18	3.27	0.003874	0.02096
	L Anterior Prefrontal Cortex (BA 10)	0.15	3.08	0.005970	0.029148
	L Dorsolateral Prefrontal Cortex (BA 46)	0.14	2.87	0.009470	0.043669
R Primary Visual Cortex (right BA 17)	R Secondary Visual Cortex (BA 18)	1.02	12.70	0.00	0.00
	L Secondary Visual Cortex (BA 18)	0.66	11.48	0.00	0.00
	L Primary Visual Cortex (BA 17)	0.54	7.59	0.00	0.000007
	R Cingulate Cortex (BA 30)	0.29	7.25	0.000001	0.000011
	R Associative Visual Cortex (BA 19)	0.48	7.13	0.000001	0.000011
	L Cingulate Cortex (BA 30)	0.16	5.93	0.000008	0.000117
	L Associative Visual Cortex (BA 19)	0.29	4.31	0.000341	0.004039
	R Fusiform Gyrus (BA 37)	0.20	3.71	0.001400	0.014520
	R Temporopolar Area (BA 38)	0.20	3.35	0.003171	0.027449
	L Dorsolateral Prefrontal Cortex (BA 46)	0.14	3.33	0.003307	0.027449
R Secondary Visual Cortex (right BA 18)	R Associative Visual Cortex (BA 19)	0.84	17.11	0.00	0.00
	L Primary Visual Cortex (BA 17)	0.66	14.83	0.00	0.00
	L Secondary Visual Cortex (BA 18)	1.02	13.96	0.00	0.00
	R Primary Visual Cortex (BA 17)	1.02	12.70	0.00	0.00
	R Cingulate Cortex (BA 30)	0.47	11.77	0.00	0.00
	L Associative Visual Cortex (BA 19)	0.56	10.82	0.00	0.00
	L Cingulate Cortex (BA 30)	0.33	9.34	0.00	0.00
	R Dorsal Posterior Cingulate Cortex (BA 31)	0.22	6.08	0.000006	0.000063
	R Fusiform Gyrus (BA 37)	0.28	5.82	0.000011	0.000099
	L Dorsal Posterior Cingulate Cortex (BA 31)	0.20	4.56	0.000189	0.001566
	L Orbitofrontal Cortex (BA 11)	0.20	4.36	0.00300	0.002248
	L Dorsolateral Prefrontal Cortex (BA 46)	0.16	4.33	0.000325	0.002248
	R Anterior Prefrontal Cortex (BA 10)	0.26	3.84	0.001015	0.006481
	L Anterior Prefrontal Cortex (BA 10)	0.18	3.67	0.001521	0.009019
	R Orbitofrontal Cortex (BA 11)	0.21	3.47	0.002413	0.013354
	L Retrosplenial Cingulate Cortex (BA 29)	0.13	3.18	0.004756	0.024672
	R Angular Gyrus (BA 39)	0.13	2.90	0.008857	0.042411
	R Ventral Posterior Cingulate Cortex (BA 23)	0.11	2.88	0.009198	0.042411
	R Piriform Cortex (BA 27)	0.16	2.79	0.01196	0.048910

*ROI* region of interest, *BA* Brodmann area, *FDR* false discovery rate, *L* left, *R* right

#### Correlations between BDI–II scores and functional connectivity during stories listening in the selected ROIs

**Fig. 2:**
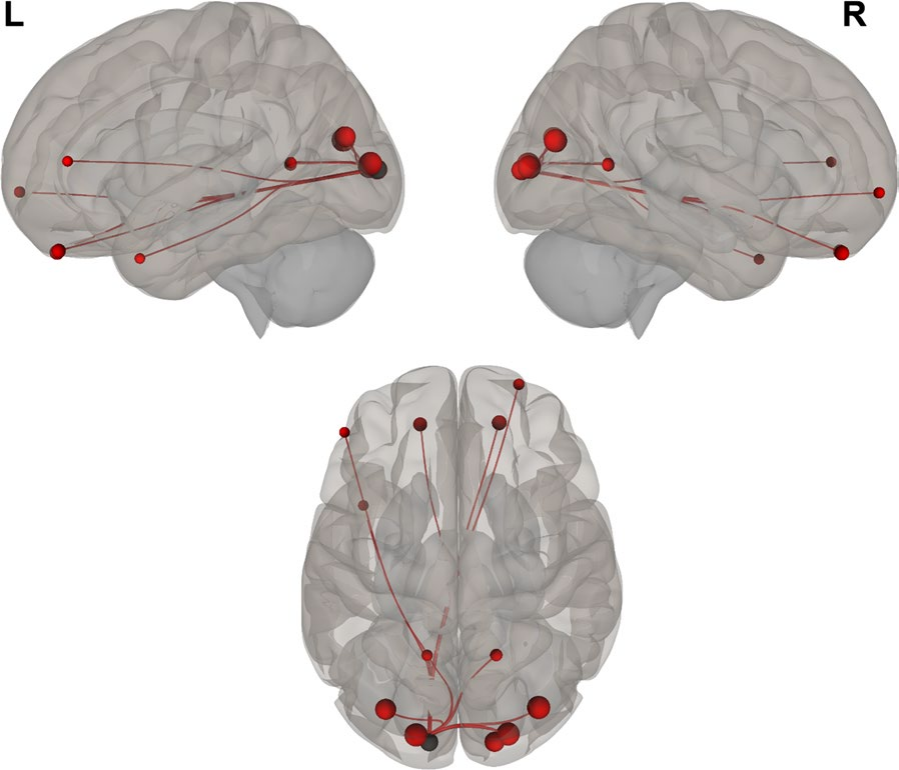
Left, right, and superior views of seed-to-region of interest (ROI) functional connectivity of left Brodmann area (BA) 17. Left BA 17 seed is shown in gray. Significant target ROIs are shown in red. Size of ROIs corresponds to effect size. *L* left, *R* right

**Fig. 3:**
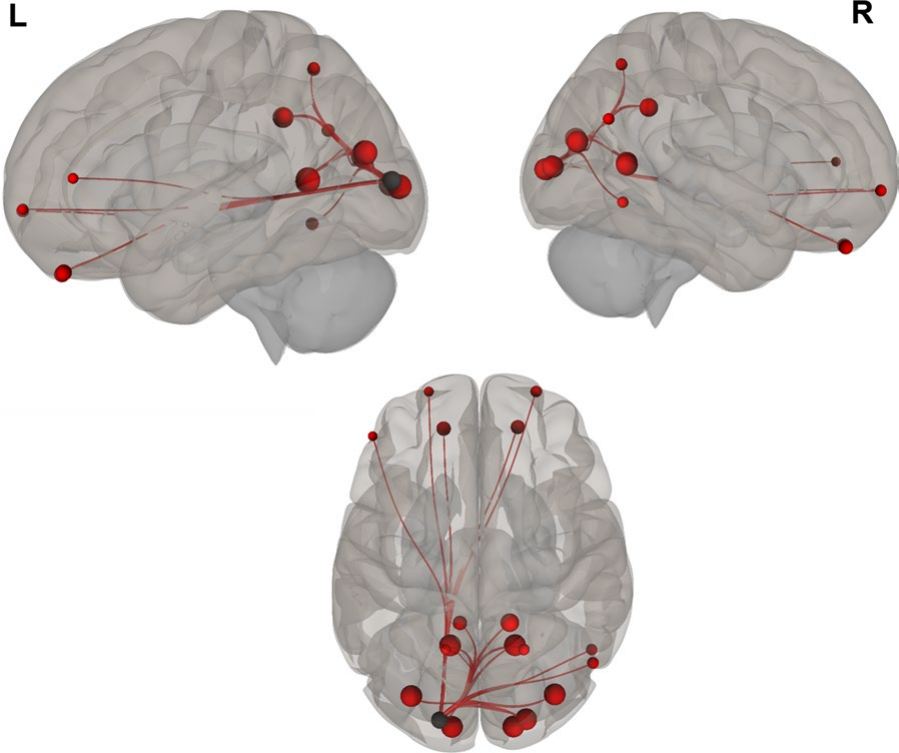
Left, right, and superior views of seed-to-region of interest (ROI) functional connectivity of left Brodmann area (BA) 18. Left BA 18 seed is shown in gray. Significant target ROIs are shown in red. Size of ROIs corresponds to effect size. *L* left, *R* right

**Fig. 4:**
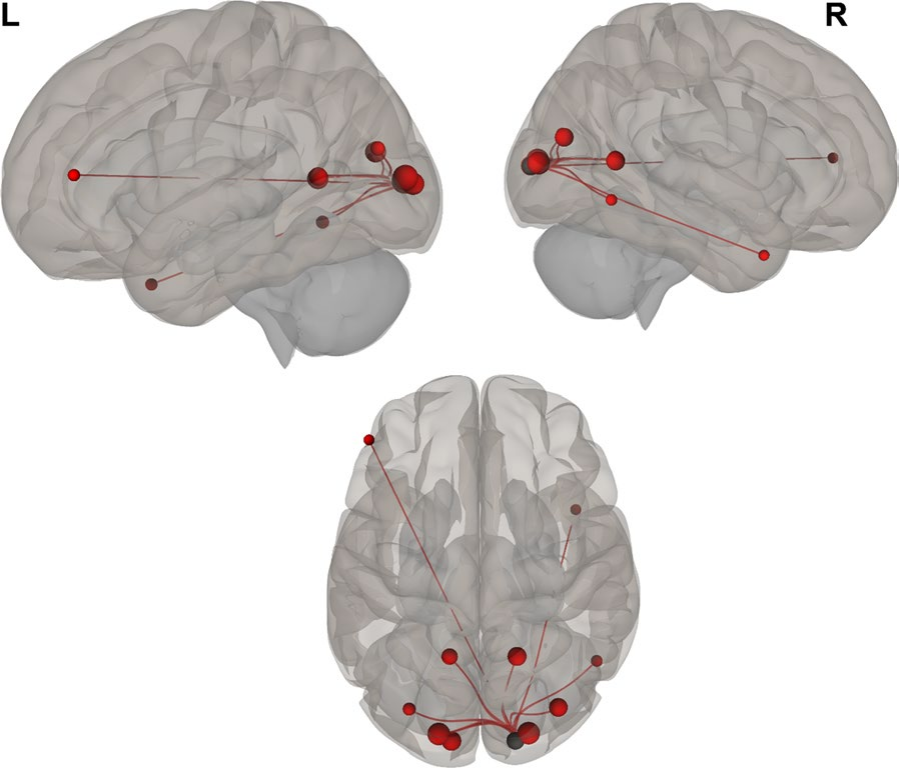
Left, right, and superior views of seed-to-region of interest (ROI) functional connectivity of right Brodmann area (BA) 17. Right BA 17 seed is shown in gray. Significant target ROIs are shown in red. Size of ROIs corresponds to effect size. *L* left, *R* right

**Fig. 5:**
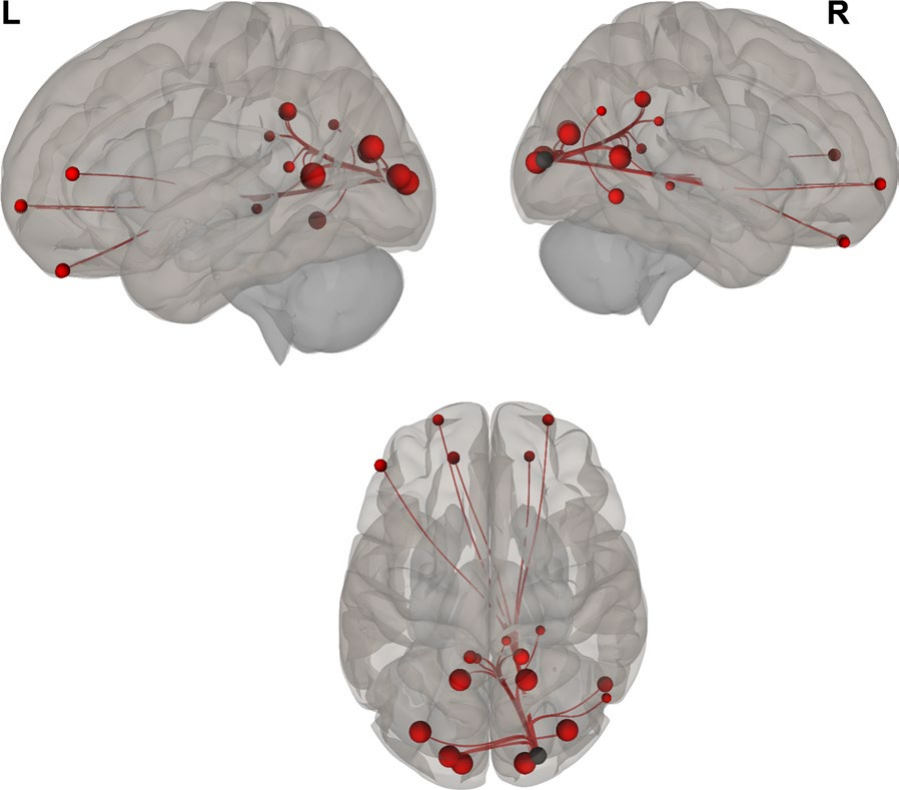
Left, right, and superior views of seed-to-region of interest (ROI) functional connectivity of right Brodmann area (BA) 18. Right BA 18 seed is shown in gray. Significant target ROIs are shown in red. Size of ROIs corresponds to effect size. *L* left; *R* right

**Fig. 6:**
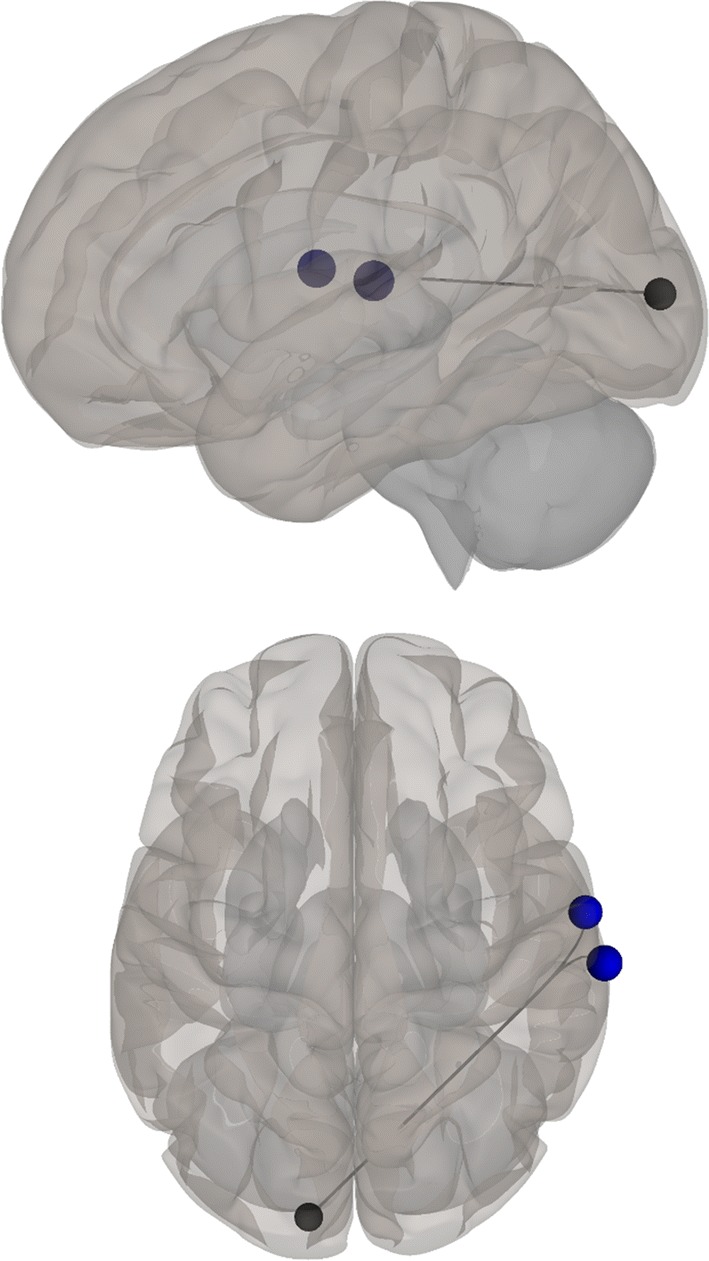
Left and superior views of the decreased functional connectivity between the left primary visual cortex [Brodmann area (BA) 17, gray] and the right primary auditory cortex (BA 42) and right subcentral area (BA 43) related to mothers’ BDI–II scores

**Fig. 7:**
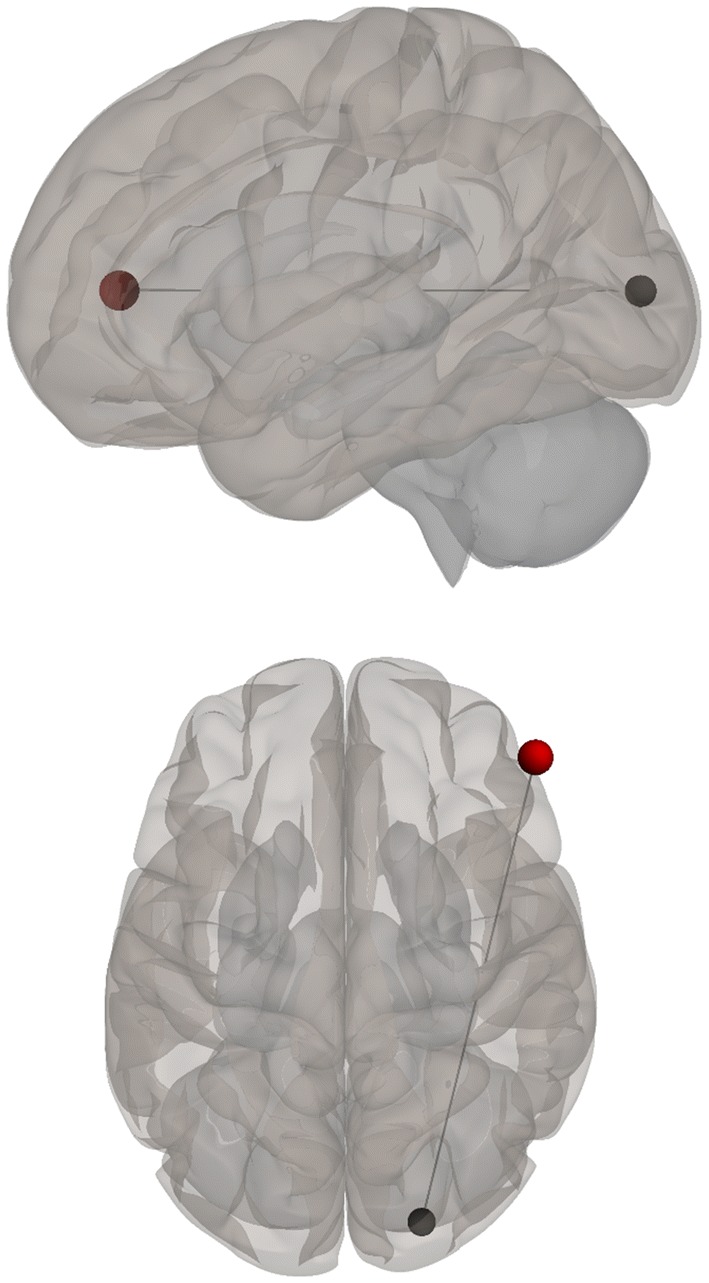
Left and superior views of the increased functional connectivity between the right secondary visual cortex [Brodmann area (BA) 18, gray] and right dorsolateral prefrontal cortex (BA 46) related to maternal depression scores

Negative correlations between BDI scores and functional connectivity when listening to stories were observed between visual (left BA 17) and auditory (right BA 42) and language (right BA 43) regions; i.e., higher depression levels were related to decreased functional connectivity between visual and auditory and language regions. Additionally, a positive correlation was found between BDI scores and functional connectivity during the stories-listening task between visual (right BA 18) and cognitive control (right BA 46) regions; i.e., higher depression levels were associated with increased functional connections between visual and cognitive control regions. Overall, greater depression level was related to a decreased association between visual and auditory regions and increased reliance of cognitive control for visualization (see Table 3 and Figs. 2, 3, 4, 5, 6 and 7).

**Table 3:** Functional connectivity between visual regions during stories listening and maternal depression score

Seed	Target (ROI)	Beta	T-value	P-value uncorrected	P-value FDR correction
L Primary Visual Cortex (BA 17)	R Primary Auditory Cortex (BA 42)	− 0.02	− 4.07	0.000653	0.047668
	R subcentral area (BA 43)	− 0.02	− 3.82	0.001149	0.047668
L Secondary Visual Cortex (BA 18)					
R Primary Visual Cortex (BA 17)					
R Secondary Visual Cortex (BA 18)	R Dorsolateral Prefrontal Cortex (BA 46)	0.02	6.12	0.000007	0.000578

*ROI* region of interest, *BA* Brodmann area, *FDR* false discovery rate, *L* left, *R* right

## Discussion

The aim of the current study was to examine the relationship between cumulative maternal depression and the development of neurobiological networks for language processing in the child. We chose to specifically examine functional connections within the visual cortex, a region related to imagination during storytelling, while the child is listening to stories. Per our hypothesis, decreased functional connections between seeds in the visual cortex and auditory processing regions were found in relation to elevated maternal depression levels. Interestingly, these relations were contralateral; i.e., data showed decreased functional connections between right visual regions and left auditory regions and between the left visual regions and right auditory regions. In support of our hypothesis, increased functional connections between the visual cortex and frontal regions were found to be associated with maternal depression; however, this was found only in the right hemisphere. Some possible explanations for this lateralization will be discussed.

### Increased maternal depression is related to decreased functional connections between visualization and auditory regions while listening to stories

Imagination is one of the critical components when listening to stories [36]. This finding, together with auditory processing previously observed during stories listening (BA 41 in 5–7 year-old children and bilateral activation in BA 41 in 11-year-old children [33]), highlight the critical role of both visual and auditory regions when listening to stories. Children who are better at comprehending stories reported that they visualize the stories they hear in their imagination, like a movie [37]. It was found that the ability to use visualization while listening to stories also supports future reading abilities in children [33]. However, the ability to visualize is not equal when listening to stories (as was required in the current study) versus reading a book or listening to a story where there is a greater visual stimulation [38]. Therefore, listening to stories requires greater attention abilities and imagination due to a lack of visual stimulation and the focus on the reader’s voice [38]. However, mothers with depression were found to read to and communicate with their children less [39, 40]. Additionally, maternal or paternal elevated depression levels in early childhood (9 and 24 months) are related to decreased reading time to their children Paulson [41]. In addition to the quantity of reading, stories reading “style” differed between depressed and non-depressed mothers, whereas depressed mothers read for a shorter time and asked fewer questions when reading the story [39, 40], though the results of this study were not compared to non-depressed mothers. Despite the lack of studies characterizing specifically the level of interaction during storytelling to their children, focusing on the child (i.e., asking children if their visualization the stories or how comfortable they feel while their mothers tell them stories) we do know that emotional regions are directly intertwined with visual processing [42]. Therefore, we submit that the decreased functional connections between the visual and auditory regions is correlated with increased maternal depression levels because the children of depressed mothers may have fewer mental resources available for visualization of the stories. These children were thought to suffer from emotional difficulties as well as negative affective states [43], which may interfere with allocating attention to visualizing the stories and enjoy the joint reading activity. A future study explicitly examining this point by asking the children if they visualized the stories, or alternatively a prospective study with scans performed at baseline and following intervention to instruct mothers how to read stories to their children dialogically would be useful to further clarify this point.

### Maternal depression is associated with greater connectivity between a child’s right visual cortex and right dorsolateral prefrontal cortex

Our study suggests a positive correlation in functional connectivity between visualization and right frontal regions (right BA 46). Although cognitive control is important in order to focus and process verbal information [44], along with occipital regions engaged in both linguistic processing and stories listening (see also [45] in children with dyslexia), the involvement of the right frontal hemisphere rather than the left frontal hemisphere, is intriguing. The activation in the right hemisphere was previously related to depression symptoms, anxiety and overall stress levels (see [46] for review). The right frontal lobe, as opposed to the left, was related to greater emotional disturbance and a lack of empathy [47]. Children of mothers with maternal depression demonstrated greater EEG electrical signal in the right frontal lobe versus the left and showed less empathy to their environment. Both infants and toddlers of depressed mothers exhibit an overall increased right frontal electrical signal versus the left hemisphere Sohr-Preston [10]. Hence, increased functional connections between the right visual region and the right frontal region may represent, in addition to the load in cognitive control during imagination while listening to stories, less positive emotional processing during the visualization process in children of mothers with elevated depression levels.

### Alternations in interhemispheric functional connections in children of depressed mothers

Our study indicates decreased functional connections between right visual and left auditory regions, as well as between left visual and right auditory regions, which raises a question regarding the relationship between maternal depression and the cross-hemispheric information transfer through the corpus callosum. Short corpus callosum was previously related to less responsive maternal behavior [48]. Kok and colleagues reviewed the literature on associations between executive function problems and a shorter corpus callosum in young children. The authors suggested that parenting style, which is related to executive functions (i.e., lack of discipline and lack of sensitivity to the child and the child’s needs), was also related to a shorter corpus callosum. It was also suggested that increased maternal depression levels may affect the development of the corpus callosum, perhaps leading to decreased functional connections between two key brain regions critical for stories listening (visual and auditory) in crossing hemispheres. However, the shorter corpus callosum described by Kok and colleagues may not be directly related to decreased interhemispheric functional activity. Therefore, using visual or auditory tasks activating one of the hemispheres and detecting the activation in the other, as related to maternal depression, may provide more accurate information related to the interhemispheric connections related to maternal depression.

### Study limitations

The results of this study may be influenced by several limitations. Our results are based on a relatively small number of participants (N = 21). Cumulative maternal depression levels at 4 years may be related to different brain activation patterns along development. A longitudinal study examining children of mothers with depression versus children of mothers without depression should be explored. To identify neural circuits related to maternal depression in children, an additional study should include a control group of non-depressed mothers. No correlations were found between BDI and child linguistic measures as examined in the current study (WIPSSI vocabulary and CTOPP phonological awareness measures). A future study should examine comprehension level and assess the correlations of those measures with BDI to strengthen the connection between comprehension and BDI. Lastly, although the focus of this study was on the relationship between maternal depression and visualization during stories listening in their children, there are additional abilities and related networks involved in this process (i.e., language and cognitive control [36, 49]). These networks may also be related to maternal depression and should be examined in depth. Last but not least, recent studies demonstrated the association between maternal depression and altered prefrontal functional connectivity in the offspring [50]. Additional studies pointed at the association between the use of antidepressant medications during the prenatal period and changes in offspring brain connectivity [51]. Hence, in the current study design it is impossible excluding the potential prenatal confounds on the current study results. This point should be clarified using longitudinal studies starting from pregnancy.

## Conclusions

The current study suggests that cumulative maternal depression levels are related to decreased functional connections between a child’s visualization regions and auditory regions that process verbal information. The reliance on frontal regions, related not only to executive functions but also to negative emotions in children, as demonstrated. These results highlight the need to monitor maternal depression during regular office visits and also even before birth since prenatal maternal depression has been associated with altered connectivity in infants [52]. Prenatal exposure and genetic depression risk may contribute to altered connectivity.

The impact of maternal depression during early childhood on neural circuitry of story listening suggests the importance of dialogic reading between mother and child. Introducing engaging reading methods to depressed mothers might improve child brain development by providing a structured context for optimal verbal stimulation and interactions with their mothers.

## Summary

The results of the current study indicate that maternal depression was correlated with decreased functional connectivity between visualization and auditory regions and increased functional connectivity in frontal regions. These findings suggest decreased imagination and increased negative emotions in children of depressed mothers and highlight the need for engaging reading methods, especially for depressed mothers and their children.

## Data Availability

Not applicable.
